# Apo-14´-Carotenoic Acid Is a Novel Endogenous and Bioactive Apo-Carotenoid

**DOI:** 10.3390/nu11092084

**Published:** 2019-09-04

**Authors:** Gamze Aydemir, Marta Domínguez, Angel R. de Lera, Johanna Mihaly, Dániel Törőcsik, Ralph Rühl

**Affiliations:** 1 Department of Biochemistry and Molecular Biology, University of Debrecen, 4032 Debrecen, Hungary; 2 Departamento de Química Orgánica, Universidade de Vigo, Faculdad de Química, 36310 Vigo, Spain; 3 Department of Dermatology, Faculty of Medicine, University of Debrecen, 4032 Debrecen, Hungary; 4 Paprika Bioanalytics BT, 4002 Debrecen, Hungary

**Keywords:** apo-carotenoids, retinoic acid, vitamin A, carotene

## Abstract

Carotenoids can be metabolized to various apo-carotenoids and retinoids. Apo-15´-carotenoic acid (retinoic acid, RA) is a potent activator of the retinoic acid receptor (RAR) in its all-*trans*- (ATRA) and 9-*cis-* (9CRA) forms. In this study we show firstly, that apo-14´-carotenoic acid (A14CA), besides retinoic acids, is present endogenously and with increased levels in the human organism after carrot juice supplementation rich in β-carotene. All-*trans*-A14C (ATA14CA) is just a moderate activator of RAR-transactivation in reporter cell lines but can potently activate retinoic acid response element (RARE)-mediated signalling in DR5/RARE-reporter mice and potently increase retinoid-reporter target gene expression in ATA14CA-supplemented mice and treated MM6 cells. Further metabolism to all-*trans*-13,14-dihydroretinoic acid (ATDHRA) may be the key for its potent effects on retinoid target gene activation in ATA14CA-treated MM6 cells and in liver of supplemented mice. We conclude that besides RAs, there are alternative ways to activate RAR-response pathways in the mammalian organism. ATA14CA alone and in combination with its metabolite ATDHRA may be an alternative pathway for potent RAR-mediated signalling.

## 1. Introduction

Carotenoids such as β-carotene are common constituents of many varieties of vegetables and fruit. The enzymatic cleavage of β-carotene to retinal is either mediated by central cleavage mechanisms or by eccentric cleavage mechanisms [1,2,3]. Retinal is further metabolized to retinol or retinoic acid. All-trans retinoic acid (ATRA) has been shown to be an endogenous activator of the retinoic acid receptors (RARs). Various physiological processes such as differentiation, apoptosis, proliferation, cell cycle, etc. are influenced by ATRA in a concentration-dependent manner (reviewed in [4]). The 9-*cis* isomer of retinoic acid has been postulated to be the endogenous activator of the retinoid-X receptor (RXR) [5,6]. Unfortunately, it is a controversial discussion whether this substance is present in sufficient physiological levels for receptor activation [7]. Recently, 9-*cis*-13,14-dihydroretinoic acid was described as an endogenous relevant RXR-activator [7,8], which is present endogenously in addition to its geometric isomer and weak RAR-agonist all-*trans*-13,14-dihydroretinoic acid (ATDHRA) [8,9,10].

Local and systemic concentration of the active endogenous RAR-agonist ATRA are well regulated by various dehydrogenases and binding proteins as well as degradation enzymes (reviewed in [4]). Systemic concentrations of ATRA are highly dependent from endogenous β-carotene concentrations and thereby dietary β-carotene uptake [11]. Low endogenous levels of ATRA might not be sufficient for explaining exclusive ATRA-mediated RAR-activation pathways and alternative RAR-specific derivatives and pathways may be of relevance. Novel retinol-derived metabolites may be of relevance like 13,14-dihydroretinoic acids [9] or lycopene-derived derivatives [12,13,14]. 

Apo-14´-carotenoic acid (A14CA) was never identified as a physiological or even nutritional-relevant retinoid/apo-carotenoid present in mammals, especially humans, and in the mammalian food matrix, while in previous studies, a natural occurrence was surprisingly claimed [15]. Organic synthesized all-*trans*-A14CA (ATA14CA) was tested in various *in vitro* systems obtaining no, weakly or questionable/non-conclusively proven, RAR-activation potential [15,16,17], and a questionable claimed RAR-antagonistic activity [15]. 

In this study we clearly identified ATA14CA as a novel endogenous apo-carotenoid/retinoid in the human body and determined it’s weak to moderate direct and indirect RAR- and RXR-transcriptional properties as well as its ability to mediate target gene expression activity *in vitro* and *in vivo*. Based on our novel knowledge, we concluded a physiological and nutritional-relevance of ATA14CA in mammals and especially humans.

## 2. Materials and Methods

### 2.1. Analytical Work

Retinoids and apo-carotenoids were determined in human serum samples by our established liquid chromatography-diode array-mass spectrometry/mass-spectrometry (LC-DAD-MS-MS) method [18] including some improvements and alterations to analyze ATA14CA and dihydroretinoic acids [8]. The detection of A14CAs has been performed using the DAD set at the maximum absorption wavelength at 378 nm using the same methodology, while MS-MS analysis for ATA14CA was shown to be less selective and sensitive in biological matrix. Isomerization of ATA14CA in DMSO solution was performed in a glass vial for 15 min under direct sunlight exposure during summer time. Sample preparation was performed using 100 μL of mouse or human serum which was diluted with a threefold volume of isopropanol, vortexed for 10 s, shaken for 6 min, and centrifuged at 13,000 rpm in a Heraeus BIOFUGE Fresco at +4 °C. After centrifugation, the supernatants were dried in an Eppendorf concentrator 5301 (Eppendorf, Hamburg, Germany) at 45 °C. The dried extracts were resuspended with 60 μL of methanol, vortexed, shaken, diluted with 40 μL of 60 mM aqueous ammonium acetate solution, and transferred into the autosampler and subsequently analyzed. The whole sample extraction procedure and HPLC-MS method has been published by Rühl [15].

### 2.2. Human Intervention Study/Recruitment and Study Design

Twenty-three non-smoking men (aged 27–40 years) of normal weight (BMI 23.1 (SD 0.39) kg/m^2^) were recruited for the study [11,19]. The study was divided into four two-week periods, resulting in a total study period of 8 weeks: weeks 1–2 low-carotenoid period, weeks 3–4 carrot juice consumption (330 mL/d 21.6 mg β-carotene, 15.7 mg α-carotene and 0.5 mg lutein; Schoenenberger, Magstadt, Germany). In order to ensure a standardized quantity of carotenoids through the vegetables, we used vegetable juices. For confirmation, a carrot juice samples was analyzed and no peak or trace of ATA14CA was detected. Subjects were told to consume the vegetable products with their main meals and to abstain from fruit and vegetables high in carotenoids throughout the whole study period. No other restrictions were placed on their daily diet. Ethical guidelines were described in the previous cited studies.

### 2.3. Reporter Cell Lines

#### 2.3.1. Preparation of Plasmids 

MH100-TK-LUC was utilized as luciferase reporter gene and β-galactosidase gene was used as internal control. RARα and RXRα constructs and β-galactosidase vector were transfected with MH100-TK-LUC. In order to equalize DNA amount, VDR-1 vector plasmid was used. All the plasmids contains ampicillin resistance gene that are controlled by SV40 promoter. DNA was transformed into *Escherichia coli* DH5-α cells using heat shock transformation. The plasmids were replicated in DH5-α *E. coli* grown in Luria Bertani (LB) medium supplemented with ampicillin (25 ng/mL). Plasmid extraction was conducted via Wizard Prep Mini Column Purification Kit (Promega, Budapest, Hungary).

#### 2.3.2. Cell Culture and Transient Transfection

Human embryonic kidney (HEK) cells were cultured in Dulbecco´s Modified Eagle Medium (DMEM) supplemented with 10% fetal bovine serum (FBS), 1% penicillin streptomycin, 2 mM L-glutamin. For experiments, 2 × 10^6^ cells were grown in T-75 flask at 37 °C with 5% CO_2_. In 24-well plates, 80,000 cells are seeded per well to obtain 70–80% confluency, 24 h before transfection. Polyethylenimine (PEI) based transfection was performed. The protocol was applied for a 24 well plate transfection. The 25 kDa PEI was obtained from Sigma/Aldrich. 1 μg plasmid DNA was diluted into 50 μL of 150 mM NaCl per well. 2 μL of PEI solution was diluted into 50 μL of 150 mM NaCl for each well. PEI solution was gently added to DNA solution and after mixing, it was incubated at room temperature for 15 to 30 min for the formation of PEI/DNA complex. DMEM supplemented with 10% FBS, 1% penicillin streptomycin, and 2 mM L-glutamin was taken out from the transfection plate and PEI/DNA complex was gently added for each well. The wells were filled with unsupplemented DMEM. Cells were transfected for 4 h, after changing the medium with supplemented (including the derivatives to be tested) DMEM, and the cells were incubated for 2 days to allow luciferase protein expression. After 48 h, cells were rinsed by 1% PBS, and lysed with reporter lysis buffer. Plates were shaken for 2 h and kept in −80 °C for 1 h. Luciferase activity of cell lysate was measured with 50 μL luciferase assay kit by luminometer (Wallac 1420 Victor). The results were normalized with β-gal assay.

ATA14CA, 9CRA, ATRA (>99% purity, were gifts from BASF AG, Ludwigshafen, Germany), LG100754, AGN193109 (gift from Ligand Pharmaceuticals, San Diego, CA, USA) were dissolved in DMSO applied to the cell culture medium.

#### 2.3.3. Reporter Mice

Retinoic acid response element luciferase construct (RARE-LUC) mice with a CD1 background [20] genetically modified to express firefly luciferase under the control of RARE (retinoic acid response element) were kindly provided by Cgene AS (Cgene AS, Oslo, Norway). Validation of the RARE-LUC system was based on a previous study [20]. The mice were housed in standard plastic cages at room temperature (20 ± 2 °C) and they had free access to both food and water. Standard pelleted laboratory mouse diet (Altromin, type VRF 1, Charles River, Budapest, Hungary) was used with the following diet composition: crude nutrients 19%, crude ash 7%, and crude fat 4.5%. Both female and male mice of 10–12 wk of age were studied. Single dose oral gavage of ATA14CA, ATRA, and control treatments (DMSO) were applied by sterilized stainless steel feeding needles 16 h before the luciferin injections and the subsequent bioluminescence imaging analyses (Table 1). All mouse experiments were approved and conducted under the guidelines and with ethical approval (25/2006 DEMAB) for the use and care of laboratory animals at the University of Debrecen, Hungary.

In all bioluminescence imaging experiments, 10–12 wk old mice were used and treated once by oral gavage 16 h before luciferin injections and bioimaging analysis. We tested organ specific expression based on bioluminescence imaging upon relatively high amounts of ATA14CA (54.3 mg/kg bw), ATRA (50 mg/kg bw), and CTRL to ensure sufficient biological activity *in vivo*. The mice (*n* = 6) were treated with 120 mg/kg D-luciferin (Promega, Budapest, Hungary) via *intra*-peritoneal injections and 5 min later anesthetized by *intra*-peritoneal (10 mg/kg) nembutal (Sigma, Budapest, Hungary) injection and 10 min later screened for whole body bioluminescence. For *ex vivo* organ analysis, mice (*n* = 6, per treatment group) were treated 15 min before killing and further organ screening with 120 mg/kg D-luciferin (Promega, Budapest, Hungary) via *intra*-peritoneal injections. After sacrificing the mice, we collected liver, spleen, lung, white adipose tissue (WAT), and intestine for bioluminescence imaging.

#### 2.3.4. Bioimaging

An Andor IQ imaging system (Andor, Belfast, Great-Britain), consisting of an Andor-ixon cooled charged coupled device (CCD) camera, housed in Unit-one (Birkerod, Denmark) black box and connected to a computer system, were utilized for data acquisition and analyses. The mice were euthanized by cervical dislocation. Subsequently, lung, WAT, liver, spleen, and intestine were rapidly excised and placed in tight light chamber for screening before freezing organs at –80 °C. The organs collected from the mice bioluminescence images were taken with 5 min integration time. Gray-scale and pseudoimages of organs were acquired by cooled CCD camera (−81 °C) and the photon signals were quantified by Andor IQ 1.6. Program. Luciferase expression was presented as integrated intensity/area. 

#### 2.3.5. MM6 Cell Culture for Target Gene Analysis

MonoMac6 cells (MM6, monocytic leukemia cells) were obtained from Sigma-Aldrich (Budapest, Hungary), stocks were stored at −70 °C prior starting the cell culture. After unfreezing MM6 cells were maintained in RPMI-1640 medium containing 10% fetal bovine serum and 5% L-glutamine, supplemented with 0.1% penicillin-streptomycin, and kept under controlled atmosphere at 37 °C and 5% CO_2_. Cell activation was achieved after two weeks. Prior to starting treatments, cells were sub-cultured every second day at a density of approximately 10^6^ cells/mL. 

Prior to plating, MM6 cells were counted by means of a Bürker chamber and centrifuged at 1000 rpm with a Jouan C312 centrifuge, and the obtained cell pellets were resuspended in RPMI-1640 medium containing 10% charcoal stripped serum and 5% L-glutamine, supplemented with 0.1% penicillin-streptomycin. Cells were adjusted to 1 × 10^6^–1.5 × 10^6^ cells/mL, and 3 mL/well were dispensed in 6-well culture dishes. Cells were incubated at 37 °C and 5% CO_2_. After 6 h various derivatives were added in an amount of 3 μL/well. Cells were incubated for 48 h at 37 °C and 5% CO_2_ and afterwards RNA extraction was performed.

#### 2.3.6. Quantitative Real Time-Polymerase Chain Reaction (QRT-PCR) 

a. In MM6 cell lines: Total RNA was isolated from cultured cells using Tri reagent solution according to the manufacturer´s instructions. Total RNA was reverse transcribed into cDNA using Super Script II First-Standard Synthesis System (Invitrogen). QRT-PCR was carried out in triplicate using Taqman probes on an ABI Prism 7900. Sequence Detector software (version 2.1, Applied Biosystems, Budapest, Hungary) was utilized for data analysis. The data are shown as mean and standard deviation values of three measurements per data point. 

b. Mouse organs PCR analysis: Beside the bioimaging experiments, qRT-PCR (quantitative Real Time-Polymerase Chain Reaction) was conducted for the analysis of mRNA expression of retinoid target genes in liver. Mouse liver was used for qRT-PCR analysis. Total RNA was isolated by Trizol® method according to the manufacturer’s directions (Invitrogen, Life Technologies Magyarorszag Kft., Budapest, Hungary). Prior to PCR total RNA samples were reverse transcribed into cDNA by enzyme according to supplier’s protocol under the following conditions: 10 min at 25 °C, 120 min at 42 °C, 5 min at 72 °C, and 10 min at 4 °C (Applied Biosystems, 2720 Thermal Cycler). qRT-PCR was performed by ABI PRISM 7900 sequence detection system (Applied Biosystems) as follows: 1 min at 94 °C, followed by 40 cycles of 12 s at 94 °C and 30 s at 60 °C. Primers were ordered from Applied Biosystems (Applied Biosystem) for mouse and probe was from ABI (Life Technologies, Budapest, Hungary). mRNA levels were normalized to the level of cyclophilin expression, which served as an internal control for the amount of RNA used in each reaction. Cycle threshold values above 40 were scored as under the limit of detection (UDL).

### 2.4. Statistical Analysis

Statistical tests for comparison of means were performed using GraphPad Prism version 5. Values are represented as mean ± SEM. For time course experiment, repeated measures of 2-way ANOVA were used to evaluate time dependent changes.

## 3. Results

**Figure 1:**
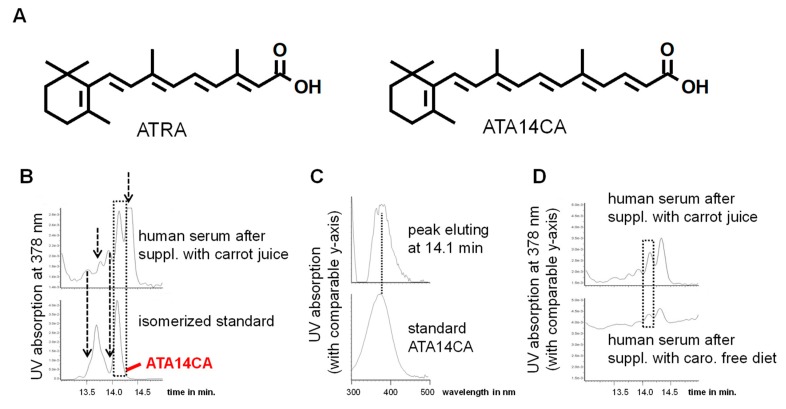
Apo-14´-carotenoic acid (A14CA) is an endogenous occurring apo-carotenoid. (**A**) Representative structural formula of all-*trans* retinoic acid (ATRA) and all-*trans*-A14CA (ATA14CA). (**B**) Representative chromatograms showing a co-elution of the all-*trans*-isomer of a photo-isomerized standard solution from ATA14CA with a peak present in human serum after supplementation with carrot juice obtaining comparable retention time of 14.1 min marked by a dashed lined box, additional unknown peaks were marked with dashed line arrows. The peaks eluting before ATA14CA obtain similar UV spectra like ATA14CA and are likely geometric isomers of ATA14CA, while the unknown peak eluting after ATA14CA obtains an UVmax of 434 nM and is likely a hydrogenation metabolite of ATA14CA. (**C**) UV spectra of the peaks eluting at 14.1 min. originating from human serum after supplementation with carrot juice and ATA14CA standard compound obtaining an UVmax of 378 nm. (**D**) Representative chromatograms from human serum after supplementation of a carotenoid free diet and after supplementation with carrot juice focusing on ATA14CA eluting at 14.1 min and marked by a dashed line box.

Identification of apo-14´-carotenoic acid (A14CA) as an endogenous apo-carotenoid/retinoid: We used a synthetic standard of all-*trans*-A14CA (ATA14CA) and isomerized it under influence of sunlight (Figure 1). The detection of A14CA was performed using a DAD detector with selected detection at 378 nm (see Figure 1A), the maximum absorption wavelength of A14CA. We determined a co-elution of the peak of ATA14CA in the chromatogram with a peak present in human serum after carrot juice supplementation. In addition, four additional peaks could be observed in human serum sample, which may be additional geometric isomers of ATA14CA and were also be present in photo-isomerized standard solution. Comparison of the UV spectra of the standard compound ATA14CA with a UVmax of 378 nm [15,21] corresponded with the UV spectra of the co-eluting compound from human serum (Figure 1B). Due to technical reasons and the availability of just a low serum sample material with low ATA14CA levels, no alternative detection techniques like MS-spectra could be performed and thereby we just have an identification of medium security. A comparison of a representative serum samples from human serum after carotenoid free diet with a serum sample from carrot juice supplementation displayed increased levels of ATA14CA. 

Further quantification shows a moderate average increase from 1.3 ± 0.6 ng/mL in human serum after carotenoid free diet to 50.5 ± 6.2 ng/mL present in human serum after carrot juice supplementation (Table 1). Additionally, ATRA levels increased in serum samples of carrot juice supplemented volunteers, while levels of the novel endogenous retinoid all-*trans*-13,14-dihydroretinoic acid (ATDHRA) remain unchanged (Table 1).

**Figure 2:**
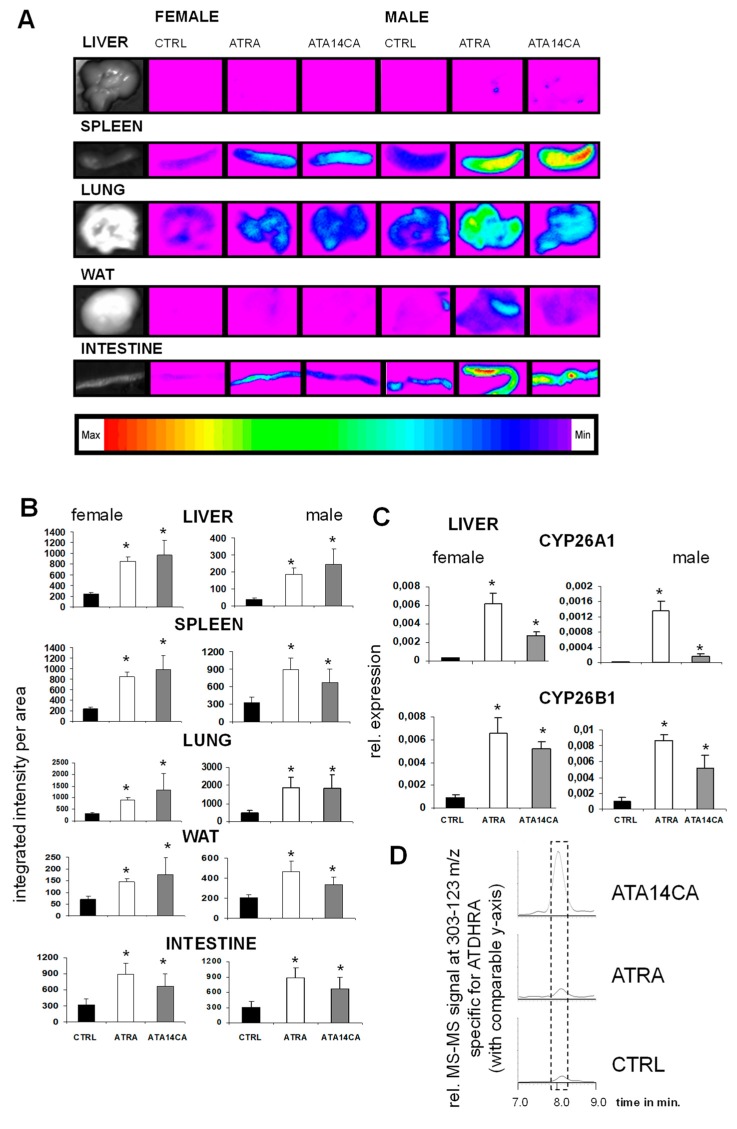
All-*trans*-apo-14´-carotenoic acid (ATA14CA) induces retinoic acid response element (RARE)-mediated signalling and RAR-target gene expression in RARE/DR5-luciferase reporter animals. (**A**) RARE/DR5-luc bioimaging of liver, spleen, lung, white adipose tissue (WAT), and intestine from male and female mice. These animals were treated with CTRL (vehicle treatment), all-*trans* retinoic acid (ATRA) (50 mg/kg bw), or ATA14CA (equimolar to ATRA-treatment with 54.3 mg/kg bw). (**B**) Quantification of the RARE/DR5-luc bioimaging data. (**C**) Quantitative real time-polymerase chain reaction (QRT-PCR) based analysis of the retinoid target genes *CYP26A1* and *CYP26B1* in liver of male and female mice. Significant values were marked with a *, when *p* ≤ 0.05 vs. CTRL. (**D**) Representative chromatograms showing increased concentrations of ATDHRA (identified using MS-MS of 303–123 m/z and marked by a dashed lined box) in mouse serum after CTRL, ATRA, and ATA14CA treatments.

ATA14CA and ATRA are equipotent in RARE/DR5-reporter animal models: ATA14CA and ATRA were administered orally in an equimolar amount to RARE/DR5-luc mice and resulted in an equipotent RARE-activation in various examined organs like liver, spleen, lung, WAT, and intestine of each compound in female as well as male animals (Figure 2A). The expression of the retinoid target genes *CYP26A1* and *B1* were both strongly up-regulated in liver samples from male and female mice (Figure 2B). Using HPLC MS-MS analysis, we identified increased levels of ATDHRA in serum samples after ATA14CA-supplemented animals, shown as representative chromatograms in Figure 2C. Serum levels of ATA14CA increased from levels under quantification limit in CRTL and ATRA supplemented animals to 756 ± 82 ng/mL, in addition to increased levels of ATDHRA from 4.6 ± 0.5 ng/mL in CTRL animals to 1455 ± 382 ng/mL in ATA14CA treated animals (Table 2). ATRA levels were both increased in ATRA treated animals (1.9 ± 0.3 ng/mL) and ATA14CA treated animals (0.9 ± 0.1 ng/mL) compared to levels which were under detection limit in CTRL-animals. 

**Figure 3:**
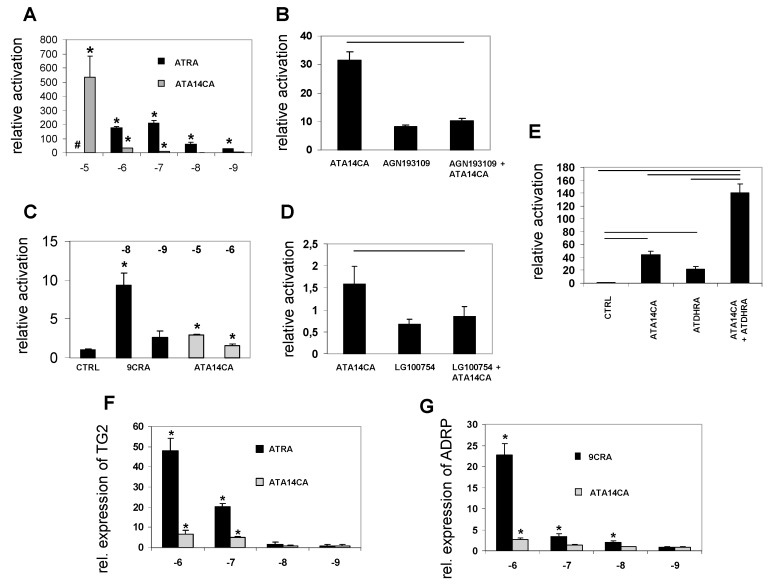
All-*trans*-apo-14´-carotenoic acid (ATA14CA) transactivates RAR- and RXR-mediated signalling and retinoid target gene expression. (**A**) Transcriptional activation of RAR-RXR heterodimer by ATRA and ATA14CA in RARα-RXRα reporter HEK cells. (**B**) RAR-antagonist AGN168109 (10^−5^ M) diminishes ATA14CA (10^−6^ M) induced RARα-mediated signalling (*p* ≤ 0.05 indicated by the black line over the bars). (**C**) Transcriptional activation of RXR heterodimer by 9CRA and ATA14CA in RXRα-reporter HEK cells. (**D**) RXR-antagonist LG100754 (10^−5^ M) diminishes ATA14CA (10^−6^ M) induced RXRα-mediated signalling (*p* ≤ 0.05 indicated by the black line over the bars). (**E**) Synergistic activation of ATDHRA (10^−5^ M) and ATA14CA (10^−5^ M) in RARα-reporter HEK cells (*p* ≤ 0.05 indicated by the black line over the bars). (**F**) ATRA and ATA14CA induce expression of the RAR target gene *TG2* in treated MM6 cells. (**G**) ATRA and ATA14CA induce expression of the RXR/PPAR target gene *ADRP* in treated MM6 cells. # ATRA at concentrations of 10^−5^ M was cytotoxic. Significant values were marked with a *, when *p* ≤ 0.05 vs. CTRL.

ATA14CA is a moderate RAR-activator and weak RXR-activator in reporter cell lines: Using reporter cell lines for RARα ATA14CA was less active than the endogenous RAR-ligand ATRA, while both derivatives were able to induce significant increased RAR-activation ranging from 10^−5^–10^−9^ M for ATRA and 10^−5^–10^−7^ M (ATRA was cytotoxic in the range of 10^−5^ M, Figure 3A). In RXR reporter cell lines ATA14CA activity was just present at levels ranging from 10^−5^–10^−6^ M. The “endogenous” RXR ligand 9CRA was highly potent and just comparable activity ranges were displayed ranging from 10^−8^–10^−9^ M, where 9CRA is significantly increasing RXR-activity at levels of 10^−8^ M (Figure 3C). ATA14CA (at 10^−6^ M) RAR as well as RXR transactivation potential was inhibited by co-application of either an RAR-antagonist AGN193168 (10^−5^ M) in RAR-reporter cell experiments (Figure 3B) or an RXR-antagonist LG100754 (10^−5^ M) in RXR-reporter cell experiments (Figure 3D). Synergistic activation of RAR-transactivation was observed by co-application of ATA14CA (10^−5^ M) and ATDHRA (10^−5^ M) (Figure 3E).

ATA14CA is a moderate activator of retinoid target gene expression: Incubation of MM6 cells with ATA14CA or ATRA ranging from 10^−6^–10^−9^ M result in significant up-regulated *TG2*-expression at 10^−6^ and 10^−7^ M, while lower levels did not significantly increase TG2 expression for both substances (Figure 3F). Increased expression of *ADRP*, an RXR-PPAR-target gene, was just observed after 10^−6^ M ATA14CA or 9CRA ranging from 10^−6^–10^−8^ M (Figure 3G). 

## 4. Discussion

All-*trans* retinoic acid (ATRA) is the most known RAR-ligand with well know nutritional and physiological relevance. In this study, we found that all-*trans*-apo-14´-carotenoic acid (ATA14CA) alone or even better in combination with its metabolite all-*trans*-13,14-dihydroretinoic acid (ATDHRA) can potently activate RAR-mediated signalling in nutritional relevant concentrations. 

ATA14CA is a low to moderate bioactive apo-carotenoid/retinoid and can induce low affinity RAR- and RXR-mediated signalling and increase RAR- and RXR-target gene expression (Figure 3 and like also shown in [15,16,17,22]) in cellular systems besides possibly acting as a potential RAR- or RXR-low affinity activators/antagonist with questionable endogenous relevance [15]. ATA14CA is here first time described as an endogenous apo-carotenoid/retinoid present in humans even after a carotenoid-wash out diet supplemented volunteers in serum levels of 1.3 ± 0.6 ng/mL (Figure 1), which are non-sufficient to initiate RAR- or RXR-mediated signalling (Figure 3A,F). Nutritional supplementation with carrot juice rich in β-carotene results in increased serum levels of 50.5 ± 6.2 ng/mL (~1.5 × 10^−7^ M). Starting at a range of 10^−7^ M serum levels after carrot juice supplementation would be sufficient to weakly activate RAR-mediated signalling (Figure 3A) like RAR-transactivation and increased RAR-target gene expression (Figure 3F), while RXR-target gene expression and RXR-transactivation were not reached by these levels (Figure 3C,G). In summary ATA14CA is a physiological retinoid/apo-carotenoid with low or unlikely any direct agonistic or antagonistic physiological- or nutritional-relevance due to its low endogenous serum levels.

**Figure 4:**
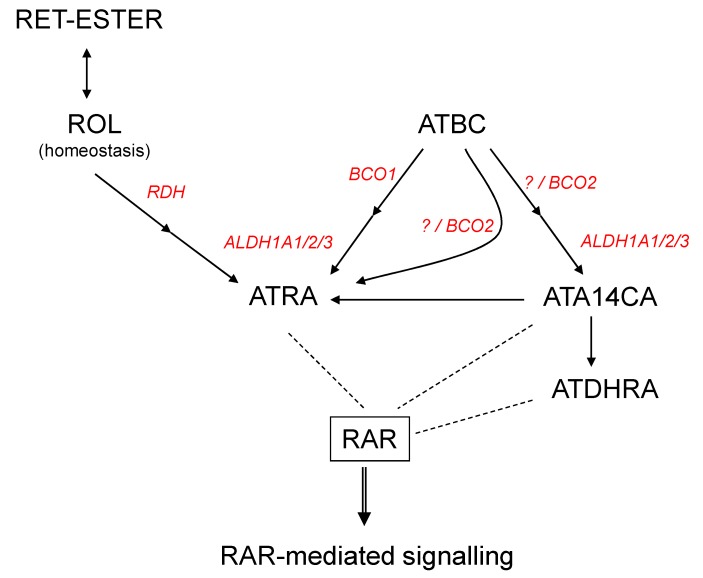
Mechanisms of all-*trans*-apo-14´-carotenoic acid (ATA14CA) mediated RAR-signalling. Scheme of retinoid and β-carotene metabolism yielding bioactive RAR-activating retinoids. Black arrows represent metabolic pathways and dashed line interaction of ligands with the nuclear hormone receptor RAR. Abbreviations: All-*trans*-β-carotene (ATBC), all-*trans* retinoic acid (ATRA), retinyl-esters (RET-ester), retinol (ROL), retinoic acid receptor (RAR), all-*trans*-13,14-dihydroretinoic acid (ATDHRA), retinol dehydrogenase (RDH), retinaldehyde dehydrogenase (ALDH1A1/2/3), beta-carotene oxygenase 1 (BCO1), beta-carotene oxygenase 2 (BCO2).

Supplementation of ATA14CA to mice resulted in increased DR5/RARE-mediated signalling comparable to equimolar administration of ATRA (Figure 2A) in higher administered amounts, which was confirmed additionally by QRT-PCR analysis of RAR-target genes in liver samples (Figure 2B). This comparable equimolar activity of ATA14CA and ATRA was explained by increased serum levels of ATDHRA after ATA14CA-administration (Figure 2C), while ATRA levels just increased minor as found previously [21]. ATDHRA was identified here as a metabolite of ATA14CA which can activate RAR-mediated signalling weakly alone or moderately in combination with its precursor-substrate ATA14CA synergistically (Figure 3E) in levels which were relevant and could be reached in human serum after nutritional carrot juice supplementation like outlined in Figure 4. 

## 5. Conclusions

ATA14CA is a novel physiological apo-carotenoid/retinoid present in humans, which likely cannot directly mediate physiological- and nutritional-relevant RAR- or RXR-mediated signalling. In combination with its metabolite ATDHRA, ATA14CA likely obtains a nutritional-relevant potential to increase RAR-mediated signalling and further retinoid dependent target gene expression *in vivo*. ATA14CA likely not alone but more likely together with ATDHRA may serve as alterative pathways for nutritional-relevant or even physiological-relevant RAR-mediated signalling besides the well-established ATRA-RAR-mediated signalling.

## Figures and Tables

**Table 1:** Concentrations of retinoids in human serum (each *n* = 23) after carotenoid free diet for 2 weeks (caro-free) and carrot juice supplementation for two weeks (carrot juice).

Diet	ATA14CA	ATRA	ATDHRA
caro-free	1.3 ± 0.6	2.7 ± 0.2	19.8 ± 1.9
carrot juice	50.5 ± 6.2 *	4.8 ± 0.2 *	24.7 ± 1.9

Presented are concentrations of all-*trans*-A14C (ATA14CA), all-*trans* retinoic acid (ATRA) and all-*trans*-13,14-dihydroretinoic acid (ATDHRA) in the serum ng/mL ± SEM. Significant values were marked with a *, when *p* < 0.05 vs. caro-free.

**Table 2:** Concentrations of retinoids in female mouse serum (each *n* = 4) of mice supplemented with vehicle (CTRL), and equimolar concentrations of all-*trans* retinoic acid (ATRA) (50 mg/kg bw) or all-*trans*-A14C (ATA14CA) (54.3 mg/kg bw) to mice.

Diet	ATA14CA	ATRA	ATDHRA
CTRL	UQL	UQL	4.6 ± 0.5
ATRA	UQL	1.9 ± 0.3 *	6.2 ± 0.4
ATA14CA	756 ± 82 *	0.9 ± 0.1 *	1455 ± 382 *

Presented are concentrations of ATA14CA, ATRA, and all-*trans*-13,14-dihydroretinoic acid (ATDHRA) in the serum ng/mL ± SEM. Significant values were marked with a *, when *p* < 0.05 vs CTRL. UQL—under quantification limit.

## References

[1] Kiefer C., Hessel S., Lampert J.M., Vogt K., Lederer M.O., Breithaupt D.E., von Lintig J. (2001). Identification and characterization of a mammalian enzyme catalyzing the asymmetric oxidative cleavage of provitamin A. J. Biol. Chem..

[2] Redmond T.M., Gentleman S., Duncan T., Yu S., Wiggert B., Gantt E., Cunningham F.X. (2001). Identification, expression, and substrate specificity of a mammalian beta-carotene 15,15′-dioxygenase. J. Biol. Chem..

[3] von Lintig J., Wyss A. (2001). Molecular analysis of vitamin A formation: Cloning and characterization of beta-carotene 15,15′-dioxygenases. Arch. Biochem. Biophys..

[4] Blomhoff R., Blomhoff H.K. (2006). Overview of retinoid metabolism and function. J. Neurobiol..

[5] Levin A.A., Sturzenbecker L.J., Kazmer S., Bosakowski T., Huselton C., Allenby G., Speck J., Kratzeisen C., Rosenberger M., Lovey A. (1992). 9-cis retinoic acid stereoisomer binds and activates the nuclear receptor RXR alpha. Nature.

[6] Heyman R.A., Mangelsdorf D.J., Dyck J.A., Stein R.B., Eichele G., Evans R.M., Thaller C. (1992). 9-cis retinoic acid is a high affinity ligand for the retinoid X receptor. Cell.

[7] de Lera A.R., Krezel W., Rühl R. (2016). An Endogenous Mammalian Retinoid X Receptor Ligand, at Last!. ChemMedChem.

[8] Rühl R., Krzyzosiak A., Niewiadomska-Cimicka A., Rochel N., Szeles L., Vaz B., Wietrzych-Schindler M., Alvarez S., Szklenar M., Nagy L. (2015). 9-cis-13,14-dihydroretinoic acid is an endogenous retinoid acting as RXR ligand in mice. PLoS Genet..

[9] Moise A.R., Kuksa V., Blaner W.S., Baehr W., Palczewski K. (2005). Metabolism and transactivation activity of 13,14-dihydroretinoic acid. J. Biol. Chem..

[10] Bazhin A.V., Bleul T., de Lera A.R., Werner J., Rühl R. (2016). Relationship Between All-trans-13,14-Dihydro Retinoic Acid and Pancreatic Adenocarcinoma. Pancreas.

[11] Rühl R., Bub A., Watzl B. (2008). Modulation of plasma all-trans retinoic acid concentrations by the consumption of carotenoid-rich vegetables. Nutrition.

[12] Aydemir G., Kasiri Y., Birta E., Beke G., Garcia A.L., Bartok E.M., Rühl R. (2013). Lycopene-derived bioactive retinoic acid receptors/retinoid-X receptors-activating metabolites may be relevant for lycopene’s anti-cancer potential. Mol. Nutr. Food Res..

[13] Gouranton E., Aydemir G., Reynaud E., Marcotorchino J., Malezet C., Caris-Veyrat C., Blomhoff R., Landrier J.F., Rühl R. (2011). Apo-10′-lycopenoic acid impacts adipose tissue biology via the retinoic acid receptors. Biochim. Biophys. Acta.

[14] Aydemir G., Kasiri Y., Bartok E.M., Birta E., Frohlich K., Bohm V., Mihaly J., Rühl R. (2016). Lycopene supplementation restores vitamin A deficiency in mice and possesses thereby partial pro-vitamin A activity transmitted via RAR-signaling. Mol. Nutr. Food Res..

[15] Eroglu A., Hruszkewycz D.P., dela Sena C., Narayanasamy S., Riedl K.M., Kopec R.E., Schwartz S.J., Curley R.W., Harrison E.H. (2012). Naturally occurring eccentric cleavage products of provitamin A beta-carotene function as antagonists of retinoic acid receptors. J. Biol. Chem..

[16] Marsh R.S., Yan Y., Reed V.M., Hruszkewycz D., Curley R.W., Harrison E.H. (2010). {beta}-Apocarotenoids do not significantly activate retinoic acid receptors {alpha} or {beta}. Exp. Biol. Med..

[17] Prakash P., Liu C., Hu K.Q., Krinsky N.I., Russell R.M., Wang X.D. (2004). Beta-carotene and beta-apo-14′-carotenoic acid prevent the reduction of retinoic acid receptor beta in benzo[a]pyrene-treated normal human bronchial epithelial cells. J. Nutr..

[18] Rühl R. (2006). Method to determine 4-oxo-retinoic acids, retinoic acids and retinol in serum and cell extracts by liquid chromatography/diode-array detection atmospheric pressure chemical ionisation tandem mass spectrometry. Rapid Commun. Mass Spectrom..

[19] Watzl B., Bub A., Brandstetter B.R., Rechkemmer G. (1999). Modulation of human T-lymphocyte functions by the consumption of carotenoid-rich vegetables. Br. J. Nutr..

[20] Aydemir G., Carlsen H., Blomhoff R., Rühl R. (2012). Lycopene induces Retinoic Acid Receptor transcriptional activation in mice. Mol. Nutr. Food Res..

[21] Wang X.D., Russell R.M., Liu C., Stickel F., Smith D.E., Krinsky N.I. (1996). Beta-oxidation in rabbit liver in vitro and in the perfused ferret liver contributes to retinoic acid biosynthesis from beta-apocarotenoic acids. J. Biol. Chem..

[22] Tibaduiza E.C., Fleet J.C., Russell R.M., Krinsky N.I. (2002). Excentric cleavage products of beta-carotene inhibit estrogen receptor positive and negative breast tumor cell growth in vitro and inhibit activator protein-1-mediated transcriptional activation. J. Nutr..

